# Assessing Population Structure and Signatures of Selection in Wanbei Pigs Using Whole Genome Resequencing Data

**DOI:** 10.3390/ani13010013

**Published:** 2022-12-20

**Authors:** Wei Zhang, Linqing Liu, Mei Zhou, Shiguang Su, Lin Dong, Xinxin Meng, Xueting Li, Chonglong Wang

**Affiliations:** 1 Key Laboratory of Pig Molecular Quantitative Genetics, Anhui Academy of Agricultural Sciences, Hefei 230031, China; 2 Anhui Provincial Key Laboratory of Livestock and Poultry Product Safety Engineering, Hefei 230031, China; 3 Institute of Animal Husbandry and Veterinary Medicine, Anhui Academy of Agricultural Sciences, Hefei 230031, China

**Keywords:** Wanbei pig, Asian wild boar, selection signatures, fixation index (FST), polymorphism levels (θπ), whole genome resequencing

## Abstract

**Simple Summary:**

The aim of this study was to perform whole-genome resequencing on Wanbei pigs and combine its data with Asian wild boar sequencing data to assess their population structure and selection signatures. A total of 176 genes were identified as selected genes that are associated with lipid metabolism, backfat thickness, muscle, and reproduction. Genomic information may play a vital role in improving conservation strategies.

**Abstract:**

Wanbei pig (WBP) is one of the indigenous pig resources in China and has many germplasm characteristics. However, research on its genome is lacking. To assess the genomic variation, population structure, and selection signatures, we resequenced 18 WBP for the first time and performed a comprehensive analysis with resequenced data of 10 Asian wild boars. In total, 590.03 Gb of data and approximately 41 million variants were obtained. Polymorphism level (θπ) ratio and genetic differentiation (fixation index)-based cross approaches were applied, and 539 regions, which harbored 176 genes, were selected. Functional analysis of the selected genes revealed that they were associated with lipid metabolism (*SCP2*, *APOA1*, *APOA4*, *APOC3*, *CD36*, *BCL6*, *ADCY8*), backfat thickness (*PLAG1*, *CACNA2D1*), muscle (*MYOG*), and reproduction (*CABS1*). Overall, our results provide a valuable resource for characterizing the uniqueness of WBP and a basis for future breeding.

## 1. Introduction

The abundance of indigenous pig resources is essential for the diversified development of animal husbandry in China and is an important constituent of the agricultural economy. Since the domestication of wild boars ~10,000 years ago [1], approximately 300 pig breeds have been globally generated with continuous natural and artificial selection [2]. Persistent selection has changed phenotypes and reshaped the genomes of pigs compared to those of their wild counterparts, including their morphology, physiology, and behavior. Unraveling the genetic basis behind selection has attracted countless biologists. With the development of sequencing technology, decrease in sequencing price, and improvement of bioinformatics, many studies have explored evolution and targeted selection to elucidate the underlying genetic mechanisms of different morphology, physiology, and behavior of pigs and provided novel insights for further improvement of pigs.

A novel variant that is under selection usually shows a high population frequency and long-range linkage disequilibrium [3]. Geneticists have proposed a series of methods to identify genes under selection [4,5,6]. Several genes associated with fertility, resistance, and meat quality have been identified in pigs. In terms of reproductive performance, several studies based on selection signature analysis in Taihu pig have identified candidate genes that are responsible for its high fertility [7,8,9]. In terms of resistance, Li et al. assembled the Tibetan pig genome and conducted a selection analysis on Tibetan and Duroc pigs to elucidate the high-altitude adaption of Tibetan [10]. The analysis of cold tolerance of the Min pig revealed that transient receptor potential cation channel subfamily V member 5 (*TRPV5*) was selected and could be a responsible gene candidate [11]. In terms of meat quality, the genetic basis of high intramuscular fat (IMF) content in Laiwu pig has been identified via whole genome resequencing [12,13]. These studies indicate that the identification of selection signatures in pigs is important for pig breeding. Although many studies have revealed causal genes for important economic traits in various pig breeds, owing to the abundant resources, more efforts are needed to elucidate the germplasm characteristics obtained from selections.

Wanbei pig (WBP), a precious Chinese autochthonous breed, is mainly distributed in Anhui Province, China. It is distinguished by its strong disease resistance, high fertility, good maternal stability, excellent meat quality, and crude-feed tolerance. Moreover, it is best favored by people in the Wanbei region. In the 1950s, the number of WBP reached 90,000. However, with the introduction of commercial pigs in the 1970s, lack of effective protection, and African Swine Fever, the number of WBPs fell from 90,000 in the 1950s to 1300 in 2009 and then decreased to 364 in 2019. Based on its small population, the WBP is at risk of extinction. In our previous study, the germplasm characteristics of WBP were investigated [14]. In recent years, increasing attention has been paid to the protection of indigenous pig breeds. The state has effectively protected the genetic resources of local pig breeds in China by building indigenous breed-protected farms, protected areas, and gene banks. However, the underlying genetic mechanism of WBP characteristics, particularly at the genome level, remains largely unknown.

Therefore, the aims of the present study were (1) to perform whole genome resequencing of the WBPs for single nucleotide polymorphism (SNP) identification, (2) to conduct a preliminary study on population structure analysis and reveal the relationship between WBPs and Asian wild boars (AWBs), (3) to elucidate the selection signatures of WBPs when compared to those of AWBs with fixation index (F_ST_) and nucleotide diversity (θπ) ratio, and (4) to annotate the selected genes for identifying genes associated with important traits, possibly reveal the excellent characteristics of local pigs, and provide a basis for the protection and development of native pigs. To a certain degree, our results provide insights and increase the understanding of the genetic basis that determines the unique traits of WBPs, presenting a scientific foundation for their protection and utilization.

## 2. Materials and Methods

### 2.1. Ethics Statement

All animal work was carried out according to the approved guidelines established by the Ministry of Agriculture of China. This study was conducted in accordance with and was approved by the Animal Care Committee of the Anhui Academy of Agricultural Sciences (Hefei, China; no. AAAS2020-04).

### 2.2. Animals and Whole-Genome Resequencing

A total of 18 unrelated WBPs (♀ = 9, ♂ = 9) of approximately 2 years old were used in this study, and they were collected from the nucleus population of the WBP population on a conservation farm in Yingshang County, Anhui Province, China. Genomic DNA was extracted from the ear samples using the standard phenol–chloroform method [15]. Isolated DNA was analyzed using a Nanodrop spectrophotometer (Thermo Fisher Scientific, Waltham, MA, USA) and 0.5% agarose gel. Subsequently, DNA samples containing more than 1.5 µg were used to build libraries, the genomic DNA of each sample was randomly fragmented, and DNA fragments of the desired length were purified. After adapter ligation and DNA cluster preparation, the fragments were sequenced on an Illumina NovaSeq 6000 platform (Illumina, San Diego, CA, USA) using paired-end 150 bp reads through Novogene (Beijing, China).

To detect the population structures and selection signatures of WBPs compared to those of AWBs, sequenced data of six AWBs were obtained from our previous study [16] and those of four AWBs were downloaded from the National Center for Biotechnology Information (NCBI) with accession numbers SRS387324, SRS387323, ERR173222, and ERR173220 [10,17].

### 2.3. Read Mapping and Single Nucleotide Polymorphism (SNP) Calling

In the process of read mapping, we removed adapters and low-quality reads using the NGSQC Toolkit [18]. The generated clean data were mapped onto the pig reference genome (Sscrofa11.1) using BWA-MEM with the default parameters [19]. We then sorted Bam files, removed duplicated reads, and calculated mapping statistics (coverage of depth) using SAMtools (https://github.com/samtools/samtools/releases/ (accessed on 12 September 2022)) [20] and Picard-tools-1.105 software (https://github.com/broadinstitute/picard/releases (accessed on 20 September 2022)). SNP calling was mainly implemented using the “HaplotypeCaller” and “VariantFiltration” modules of the GATK software based on the alignment files. To obtain high-quality SNP in this study, the following criteria were used: variant confidence/quality by depth (QD) < 2.0, RMS mapping quality (MQ) < 40.0, Phred-scaled *p* value (FS) using Fisher’s exact test to detect strand bias >60.0, Strand Odd Ratio (SOR) > 3.0, Z-score (MQRankSum) from Wilcoxon rank sum test of Alt vs. Ref read mapping qualities < −12.5, Z-score (ReadPosRankSum) from Wilcoxon rank sum test of Alt vs. Ref read position bias < −8.0, and cluster 2 window 5. The filtered SNPs were annotated using ANNOVAR based on gene-based and region-based models [21]. The downloaded resequenced data were analyzed using the process described above.

### 2.4. Population Structure and Linkage Disequilibrium Analysis

To investigate the population structure and selection signatures between the WBP and AWB populations, we filtered all autosome SNPs with minor allele frequency <0.05, Hardweinberg equilibrium test *p* value < 1 × 10^−6^, and call rate ≥ 90%. The VCF file of the SNP was then converted to PLINK input file formats (map and. ped) using PLINK software v.1.90. Principal component analysis (PCA) was performed using GCTA v.1.25 [22] to show the clustering of individuals and understand the genetic structure of the population. Neighbor-joining (NJ) phylogenetic trees were constructed based on the identical-by-state distance matrix using the PHYLIP v.3.695 [23] package to infer their kinship distance and reveal the evolutionary relationships among populations. ADMIXTURE v1.3.0 [24] was used to analyze the population genetics and reveal the evolutionary process. The genome-wide linkage disequilibrium (LD) pattern between the WBP and AWB populations was assessed using PopLDdecay software (https://github.com/BGI-shenzhen/PopLDdecay (accessed on 8 October 2022)) with default parameters.

### 2.5. Identification of Selection Signatures

The fixation index (F_ST_) is a measure of population differentiation and genetic distance [25]. Nucleotide diversity (θπ) refers to the mean number of SNP differences between any two different individuals in the population. The θπ- and F_ST_-based cross approaches were conducted to identify the selection signatures in WBPs compared to those of AWBs using a 100-kb sliding window approach with a 10-kb step size in PopGenome [26]. The overlapping regions within the top 1% of the F_ST_ and θπ ratios were considered selection signatures. Finally, genes in the selection signatures were retrieved from the BioMart software (http://asia.ensembl.org/biomart/martview/ (accessed on 1 November 2022)) and analyzed for functional enrichment of Gene Ontology (GO) and Kyoto Encyclopedia of Genes and Genomes (KEGG) pathways based on Sscrofa11.1 using KOBAS (http://kobas.cbi.pku.edu.cn/, accessed on 6 November 2022). The statistical method used was the hypergeometric test/Fisher’s exact test, and the correction method was the Benjamini and Hochberg method (1995). The terms and pathways that exhibited corrected *p*-values < 0.05 were considered significant.

## 3. Results

### 3.1. Sequencing and Identification of SNPs

Whole genome resequencing of WBP (*n* = 18) yielded 590.03 Gb data with an average depth of 12.61 (Table S1). The data were jointly analyzed with sequenced data from 10 AWBs, resulting in 844.35 Gb data (Table S2). After alignment with the reference genome and variant calling, approximately 41 million SNPs were identified in the WBP population. The average transition/transversion ratio and heterozygosity/homozygosity were 2.44 and 2.41, respectively, corroborating the results of a previous study [27]. Further functional annotation of the SNPs in WBP revealed that 53.32% of SNPs were found in intergenic regions, 43.26% were found in intronic regions, 1.5% were located in untranslated regions (UTR), 1.1% were observed upstream and downstream of genes, and only 0.72% were located in the coding sequences (Table S3).

### 3.2. Population Structure and Linkage Disequilibrium Analysis

**Figure 1:**
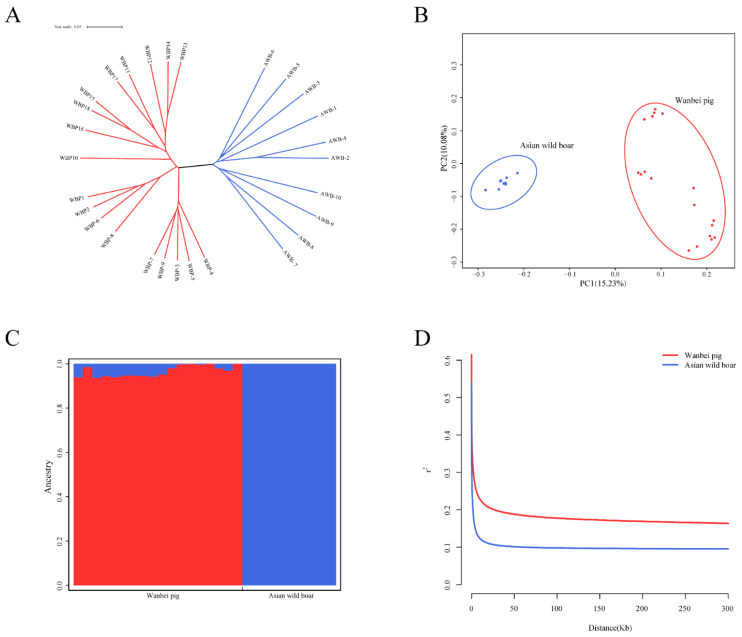
Population structure and LD analysis. (**A**) Neighbor-joining tree constructed from SNP data among study population. (**B**) Principal component plots for the first two PCs for all 28 individuals. (**C**) Structure analysis on all the AWB and WBP with K = 2. (**D**) Correlation coefficients (r^2^) were calculated for the AWB and WBP over 50-kb windows.

To assess the population structure and linkage disequilibrium of WBPs and AWBs, we used the hard filtering criteria described in Section 2. After filtering, 7,527,429 SNPs were used to conduct population structure and linkage disequilibrium analyses. We first examined the NJ tree of WBPs and AWBs and found that the two populations formed their own separate clusters (Figure 1A). Second, we performed PCA analysis and found that WBPs and AWBs were effectively separated. PC1 and PC2 explained approximately 15.23% and 10.08% of the total genetic variation, respectively (Figure 1B). Third, K = 2 was used to assess the degree of mixture in the two populations. The results were also verified with PCA and NJ trees (Figure 1C). Moreover, analysis of the LD of WBP and AWB populations revealed that the overall trend was similar to that of the increase in distance (Figure 1D). A lower LD decay was observed in the WBP population than in the AWB population, indicating that the selection caused the enhancement of LD degree in WBPs.

### 3.3. Candidate Genes under Selection Signatures

**Figure 2:**
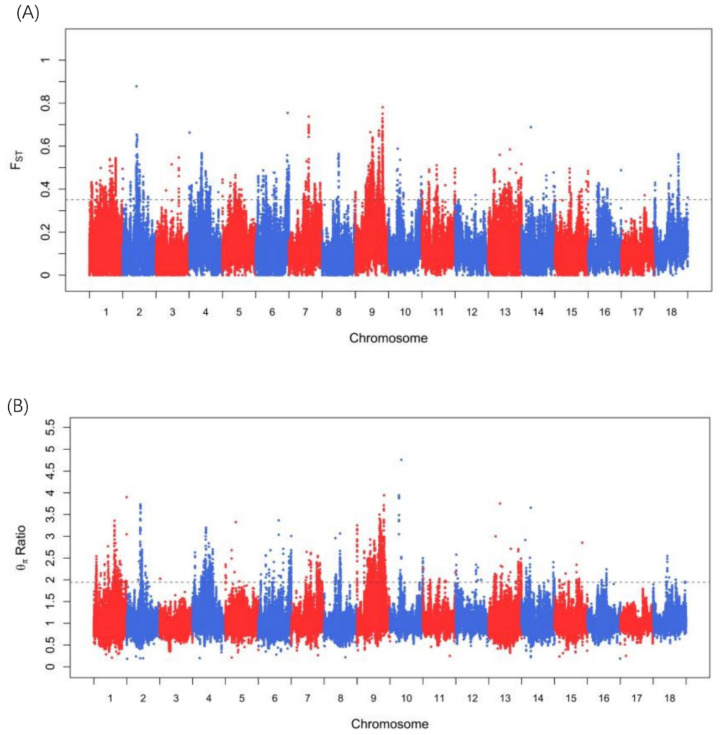
Identification of genomic regions with selection in the Wanbei pig population compared to Asian wild boar, which are calculated in a 100-kb sliding window approach with 10 kb step-size. (**A**) Distribution of FST values among autosomal chromosomes. The grey line represents the 0.01 level. (**B**) Distribution of θπ ratio among autosomal chromosomes. The grey line represents the 0.01 level.

F_ST_ and θπ ratio-based cross approaches were employed to identify the selection signatures in the WBP breed compared to those in the AWB population across the autosome. In this study, only a region that was within the top 1% of the F_ST_ and θπ ratio could be identified as a selected region. A total of 2246 regions were identified in the top 1% of the two statistics (threshold: 1%, F_ST_: 0.3507; θπ ratio: 1.944; Tables S4 and S5). The Manhattan plot of the two statistics across autosomes is shown in Figure 2A,B. After combining the two statistics, we found 539 regions (53.9 Mb of the genome, Table S6). In total, 176 genes were identified in the selected regions (Table S7). To assess the function of the selected genes, GO terms and KEGG pathways were determined using KOBAS with a corrected *p*-value of less than 0.05 as significant. In the GO analysis, a total of 65 terms were significantly enriched (Figure S1, Table S8), including lipoprotein metabolic process (GO:0042157, corrected *p* = 0.0128722, *APOA1*, *APOA3*, and *APOA4*), cholesterol homeostasis (GO:0042632, corrected *p* = 0.0238305, *APOA1*, *APOA3*, *APOA4*, and *NR5A2*), activation of MAPK activity (GO:0000187, corrected *p* = 0.0238305, *HGF*, *ERP29*, *PTPRC*, and *MOS*), negative regulation of inflammatory response (GO:0050728, corrected *p* = 0.0238305, *HGF*, *ADORA1*, *APOA1*, and *LRFN5*), regulation of CDC42 protein signal trans duction (GO:0032489, corrected *p* = 0.0238305, *APOA1* and *APOA3*), and positive regulation of fatty acid biosynthetic process (GO:0045723, corrected *p* = 0.0256596, *APOA1* and *APOA4*). In the KEGG analysis, six pathways were significantly enriched (Figure S2, Table S9), including PPAR signaling pathway (ssc03320, corrected *p* = 0.01372476, *SCP2*, *CYP4A24*, *APOA1*, *CD36*, and *APOC3*), cholesterol metabolism (ssc04979, corrected *p* = 0.020822056, *APOA1*, *APOA4*, *CD36*, and *APOC3*), relaxin signaling pathway (ssc04926, corrected *p* = 0.028051625, *COL1A2*, *ADCY8*, *RLN2*, *GNGT1*, and *GNAI1*), and fat digestion and absorption (ssc04975, corrected *p* = 0.032326776, *APOA1*, *APOA4*, and *CD36*).

## 4. Discussion

Pig breeds are an important strategic resource for ensuring the safe and sustainable development of the pig industry. Pigs play an important role in China’s national economy, accounting for approximately 60% of the meat consumption by Chinese residents. With the improvement of living standards, the demand for high-quality pork is rapidly increasing. China has abundant local pig genetic resources, and these native pig breeds generally have a good meat quality. However, the lack of systematic and comprehensive research on the characteristics of germplasm resources has led to a serious shortage in the exploitation and utilization of the excellent characteristics of local breeds, which has restricted the protection, development, and utilization of local genetic resources and limited the competitiveness of the market and ability to sustainably develop the industry. To better understand the characteristics of the germplasm in WBPs, we sequenced 18 unrelated WBPs and obtained the whole genome variations. Afterward, 10 AWBs were combined to analyze the population structure and selection signatures. A total of 539 regions were identified, and 176 genes were identified in the selected regions. Functional enrichment analysis revealed that the genes were related to lipoprotein metabolic process, activation of MAPK activity, negative regulation of inflammatory response, regulation of CDC42 protein signal transduction, positive regulation of fatty acid biosynthetic process, PPAR signaling pathway, cholesterol metabolism, relaxin signaling pathway, and fat digestion and absorption.

**Figure 3:**
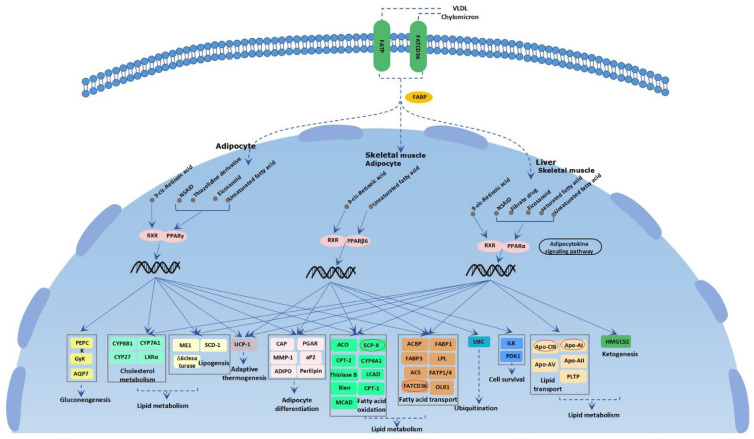
The overview of the PPAR signaling pathway. The function of the *SCP2*, *APOA1*, *CD36*, and *APOC3* are described in the figure and marked with red ellipses.

Several genes were found to be related to lipid metabolism. Sterol carrier protein (*SCP2*), known as the nonspecific lipid transfer protein, plays an important role in lipid metabolism [28,29]. McLean et al. (1995) suggested that *SCP2* expression levels are altered in several diseases in which lipid metabolism is abnormal [30]. In yaks, the expression level of *SCP2* was significantly associated with C10:0, C12:0, and C14:0 (*p* < 0.05) in the longissimus dorsi muscle, indicating that it participates in the regulation and control of intramuscular fatty acid metabolism [31]. *SCP2* was found to be a selected gene in Jeju Black cattle and is associated with meat quality [32]. Apolipoprotein A1 (*APOA1*), mainly synthesized by the liver and intestine, belongs to the apolipoprotein gene family that encodes the important regulators of lipid biosynthesis and metabolism [33]. *APOA1* is also involved in cholesterol transport [34]. In chickens, single-cell RNA sequencing and proteomics of breast muscle showed that *APOA1* was associated with IMF and could be regarded as a marker gene for IMF studies [35,36]. Several studies on the potential function of *APOA1* have been conducted to date. By integrating the multisource transcriptomes of lean- and fat-type pigs using machine learning, *APOA1* was specifically expressed in the liver, suggesting it to be an important candidate biomarker for fat deposition [37]. It was revealed through iTRAQ-based proteomic analysis of two Chinese native pig breeds (Tibetan pig and Diannan Small-Ear pig) and two commercial breeds (Yorkshire and Landrace) that *APOA1* may be a key protein affecting lipid deposition in pigs [38]. In cattle and yak, Qin et al. [31] revealed that *APOA1* was positively correlated with C12:0 and C15:0 (*p* < 0.05) in yak and negatively correlated with SFA, C16:0, and C18:0 (*p* < 0.05) in cattle, suggesting that *APOA1* may be a genetic marker that could regulate fatty acid deposition. Apolipoprotein C3 (*APOC3*), a member of the apolipoprotein family, has been identified as a regulator of triglycerides and total cholesterol [39]. In transgenic mice, the expression level of *APOC3* is positively correlated with serum triglyceride concentration [40,41]. *CD36* molecule (*CD36*), a membrane glycoprotein that interacts with a large variety of ligands, plays an important role in the membrane transport of long-chain fatty acids in the heart, skeletal muscle, and adipose tissue [42]. In cattle, *CD36* was first identified to regulate IMF deposition and then regarded as a selected gene involved in fat digestion and absorption, which may elucidate the characteristics of cold resistance [43,44]. In chickens, *CD36* was found to be a selected gene involved in lipid metabolism [45]. The expression of *CD36* has been proven to be positively correlated with obesity in dairy cows [46]. In rats, *CD36* has been reported to play an important role in fatty acid oxidation in skeletal muscle [47]. *SCP2*, *APOA1*, *CD36*, and *APOC3* were all significantly enriched in the PPAR signaling pathway (https://www.cusabio.cn/pathway/PPAR-signaling-pathway.html (accessed on 12 October 2022). As shown in Figure 3, these four genes play vital roles in lipid metabolism.

Apolipoprotein A4 (*APOA4*), a member of the apolipoprotein family, is primarily synthesized by the small intestine and plays an important role in mediating reverse-cholesterol transport and participating in triglyceride absorption [48]. In chickens, a genome-wide association study of 1400 samples revealed that *APOA4* was within a QTL associated with fat deposition, indicating the potential effect of *APOA4* [49]. Meanwhile, the *APOA4* regulates triglyceride metabolism in humans and mice [33,34]. Moreover, *APOA4* is involved in gluconeogenesis and lipid metabolism in the livers of dairy cows [50,51]. In pigs, correlation analysis of proteomics and lipidomics based on extreme intramuscular fat in the Xidu pig population revealed that *APOA4* is related to triacylglycerols [52]. B-cell lymphoma 6 (*BCL6*), a zinc finger protein, is a transcription factor that was originally identified as a proto-oncogene [53]. *BCL6* was found to be associated with adipose development by regulating lipid metabolism [54]. In a study, a knockdown of *BCL6* inhibited adipogenic potential, whereas *BCL6* overexpression enhanced adipogenic differentiation, revealing that *BCL6* may be a key factor during early adipogenesis [55]. Adenylate cyclase 8 (*ADCY8*), a member of the adenylate cyclase family, plays an important role in nutrient homeostasis in rodents [56,57,58]. The *ADCY8* genes have been identified as selected genes in cattle [59]. In a genome-wide association study of serum mineral elements in 587 Chinese Han people, genetic loci in *ADCY8* were significantly associated with Mg and Fe element levels (*p* < 5 × 10^−6^) [60]. Meanwhile, the *ADCY8* gene is also involved in a metabolic pathway associated with high-density cholesterol in human cohorts [61]. In pigs, genome-wide association studies of 30 hematological and blood clinical-biochemical traits in 843 Large White pigs revealed that *ADCY8* was associated with total cholesterol and high-density lipoprotein cholesterol [62].

Some genes, which regulate back fat thickness, have also been identified. Commercial pigs, such as Large White, Landrace, and Duroc, have undergone highly intensive selection and provided more pork in the past decades, leading to their dominance in the global pig industry. In the history of pig cultivation in China, pigs not only provided meat but also provided fat to meet people’s living needs because fat was an important source of energy for Chinese farmers in ancient times; thus, Chinese native pig breeds have higher backfat thickness, with good meat quality and excellent adaptability to diverse environments compared to commercial pig breeds. Pleomorphic adenoma gene 1 (*PLAG1*), a member of the pleomorphic adenoma gene family, encodes a multifunctional transcription factor that controls many genes and pathways, such as the insulin-like growth factor (IGF)-II, *IGF-1R*, and WNT pathways [63]. Previous studies have revealed that *PLAG1* was identified as a selected gene and is associated with body size traits in humans and cattle [64,65,66,67]. Moreover, *PLAG1* can affect the onset of puberty in heifers through GH signaling and its direct effect on *IGF1* levels [68]. In pigs, *PLAG1* appears to be one of the top genes selected during the domestication of European pigs, and *PLAG1* variants have been associated with growth and fatness traits [69]. In a genome-wide association study for growth and fatness traits in Sujiang pigs, a synonymous SNP (SSC4:75 691 055, A > G, rs326013678) in *PLAG1* was found, and genotype–phenotype analysis of 365 Sujiang pigs and 150 durocs revealed that there were only AA genotypes in Duroc pigs. Moreover, the AA genotype had significantly smaller backfat thickness values than other genotypes in Sujiang pigs [70]. In this study, the *PLAG1* gene was identified and the same synonymous SNP (SSC4:75 691 055, A > G, rs326013678) was also found, corroborating the history of Chinese native pig breeding. We may conclude that the loci in *PLAG1* could be used for breeding Chinese indigenous pigs with backfat thickness. Calcium voltage-gated channel auxiliary subunit alpha 2 delta 1 (*CACNA2D1*), which is a member of the calcium voltage-gated channel auxiliary subunit alpha-2/delta, has been previously reported to be a candidate gene associated with somatic cell score and mastitis resistance [71]. The variations in *CACNA2D1* were significantly associated with backfat thickness (*p* < 0.001) [72].

Several genes associated with other economic traits were identified in this study. Myogenin (*MYOG*), a member of the well-known myogenic regulatory factor (MRF) family, plays a crucial role in myogenesis by acting synergistically to stimulate and initiate the differentiation process of myoblasts [73]. The *MYOG* has been identified as a selected gene in pig and cattle [74,75]. In a previous study, pigs with different genotypes of *MYOG* were associated with the number of muscle fibers and rate of growth, causing a variation in muscle mass [76]. In chickens, it has been suggested that *MYOG* can be used in marker-assisted selection to improve growth traits in chickens [77]. Mineral elements play important biological roles in enzymes, hormones, vitamins, and normal metabolism [78]. Deficiency in mineral elements can lead to abnormal physiological functions [79]. Calcium-binding protein, such as sperm specific 1 (*CABS1*), is specifically expressed in the elongated spermatids of mice and then localized to the principal piece of flagella of matured spermatozoa [80]. Shawki et al. found that the porcine *CABS1* is localized to acrosome in addition to the tail, where mCABS1 only localizes in mature sperm, suggesting that porcine *CABS1* is involved in acrosome reaction [81].

Although some interesting findings have been reported in this study, the limitations of this study should not be neglected. First, the number of WBPs compared with that of AWBs is small and may have affected the results of this study. However, to some extent, there is also a preliminary understanding of the differences between the two populations. Further, due to the influence of COVID-19 and swine fever, a functional verification experiment of the selected genes of interest was not carried out. The functions of the selected genes were searched in previous studies. The limitations might impact the observations of this study and should be overcome in further investigations by (i) increasing the study population and collecting samples and phenotypes and (ii) verifying the effect of variation through association analyses.

## 5. Conclusions

In this study, the genetic variation, population structure, and selection signatures of three bWBP breed in Anhui province were analyzed using the whole-genome resequencing for the first time. We also detected the selection signatures of WBPs compared with those of AWBs and discovered many genes that are associated with lipid metabolism, backfat thickness, muscle, and reproduction. Our findings will help to better manage the WBP breed and improve breeding, which is vital for protecting Chinese native pigs and promoting the development of indigenous pig breeds.

## Data Availability

The data set used and analyzed during the current study is available from the corresponding author on reasonable request.

## Supplementary Materials

The following supporting information can be downloaded at: https://www.mdpi.com/article/10.3390/ani13010013/s1, Figure S1. The GO analysis of the selected genes. Figure S2. The top 20 pathways. Table S1: The summary statistic of resequencing data in WBP. Table S2: the statistics of 10 sequenced Asian wild boar; Table S3: the percentage of variants annotation; Table S4: The information for the selected region for FST; Table S5: The information for the selected region for θπ ratio; Table S6: the information for the 539 selected regions; Table S7: the information for the 176 selected genes; Table S8: GO analysis of the selected genes; Table S9: KEGG analysis of the selected genes.
